# Genetic Properties of a Nested Association Mapping Population Constructed With Semi-Winter and Spring Oilseed Rapes

**DOI:** 10.3389/fpls.2018.01740

**Published:** 2018-11-26

**Authors:** Jianlin Hu, Chaocheng Guo, Bo Wang, Jiaqing Ye, Meng Liu, Zhikun Wu, Yingjie Xiao, Qinghua Zhang, Haitao Li, Graham J. King, Kede Liu

**Affiliations:** ^1^National Key Laboratory of Crop Genetic Improvement, Huazhong Agricultural University, Wuhan, China; ^2^Southern Cross Plant Science, Southern Cross University, Lismore, NSW, Australia

**Keywords:** rapeseed (*Brassica napus*), NAM population, genetic linkage map, GWAS, population structure, recombination

## Abstract

Nested association mapping (NAM) populations have been widely applied to dissect the genetic basis of complex quantitative traits in a variety of crops. In this study, we developed a *Brassica napus* NAM (BN-NAM) population consisting of 15 recombination inbred line (RIL) families with 2,425 immortal genotypes. Fifteen high-density genetic linkage maps were constructed by genotyping by sequencing (GBS) based on all RIL families, with further integration into a joint linkage map (JLM) having 30,209 unique markers in common with multiple linkage maps. Furthermore, an ultra-density whole-genome variation map was constructed by projecting 4,444,309 high-quality variants onto the JLM. The NAM population captured a total of 88,542 recombination events (REs). The uneven distribution of recombination rate along chromosomes is positively correlated with the densities of genes and markers, but negatively correlated with the density of transposable elements and linkage disequilibrium (LD). Analyses of population structure and principal components revealed that the BN-NAM population could be divided into three groups with weak stratification. The LD decay distance across genome varied between 170 and 2,400 Kb, with LD decay more rapid in the A than in the C sub-genome. The pericentromeric regions contained large LD blocks, especially in the C sub-genome. This NAM population provides a valuable resource for dissecting the genetic basis of important traits in rapeseed, especially in semi-winter oilseed rape.

## Introduction

Most important traits in crops are genetically determined by polygenes and interaction effects between genes and/or genes and the environment. The genetic basis of quantitative trait loci (QTLs) has conventionally been dissected by QTL mapping in segregating populations derived from biparental crosses. QTL mapping has been successful in detecting contributions from thousands of loci in many crops, and greatly facilitated the identification of many agronomically important genes and molecular marker-assisted breeding. Although this approach had proven to be efficient in detecting QTL effects at low resolution, it is often hard to identify closely linked markers for fine mapping and marker-assisted selection due to the relatively low frequency and coverage of recombination events in single or several crosses. In addition, where loci have identical alleles in both parents it is not possible to detect recombination events due to lack of segregation.

Genome-wide association studies (GWAS) have more recently been extensively used to compensate for drawbacks of the conventional QTL mapping approach. In comparison, GWAS is able to survey numerous historical recombination events in collections of landraces, varieties and breeding lines (Flint-Garcia et al., 2003), and so potentially achieve a far higher resolution of QTL mapping than was possible relying solely on biparental segregating populations. GWAS has been particularly successful in interpreting the associations between molecular markers and traits (Si et al., 2016; Yano et al., 2016), although the filter of rare variants and inherent population structure in natural populations tends to reduce statistical power (Xiao et al., 2017). Rare variants may be the cause of the phenotypic variant of interest and thus a source of the missing heritability (Eichler et al., 2010). Moreover, it is hard to detect variants underlying traits of interest correctly if they are significantly correlated to population structure (Flint-Garcia et al., 2005). To overcome spurious associations and increase the detection power of rare alleles in crop species, multi-parent cross populations using different cross designs have been developed, including Nested Association Mapping (NAM), Multi-parent Advanced Generation Inter-Crosses (MAGIC), and Random-open-parent Association Mapping (ROAM) (Yu and Buckler, 2006; Dell’Acqua et al., 2015; Xiao et al., 2016). The NAM population design was first proposed in maize (Yu and Buckler, 2006; Yu et al., 2008), and the first example in maize consisted of 5,000 RILs derived from 25 segregating families generated from crossing the homozygous B73 line with 25 lines representing a wide coverage of the domesticated maize genepool (McMullen et al., 2009). This study demonstrated that the NAM population approach was powerful in dissecting the genetic architecture of complex quantitative traits including flowering time, leaf architecture, stalk strength and plant height (Buckler et al., 2009; Tian et al., 2011; Peiffer et al., 2013, 2014; Li et al., 2016). This initial success prompted the development of NAM populations in other crops such as rice, wheat, barley, soybean, and sorghum (Maurer et al., 2015; Schmutzer et al., 2015; Bajgain et al., 2016; Bouchet et al., 2017; Fragoso et al., 2017; Song et al., 2017).

Rapeseed (canola, oilseed rape, *Brassica napus* L.) is grown in many countries for the production of high-quality edible oil, biodiesel with the protein-rich meal used as livestock feed. It is the third-leading source of vegetable oil, after soybean and palm oil, and also the world’s second-leading source of protein for meal after soybean^1^. China is the world’s largest consumer of rapeseed, but the second-leading producer in the world. Since the production of rapeseed in China can only provide about 40% of the vegetable oil supply (Sun et al., 2012), there is a large gap between rapeseed production and consumption. Development of cultivars with high yield is one of the most important ways to fill this gap, which requires the employment of modern genetics and breeding technologies to dissect the genetic basis of important traits. The genetic basis of many important rapeseed traits such as flowering time, oil content, yield-related traits have been analyzed with thousands of QTL detected by mapping in biparental segregating populations and in landraces and cultivars using GWAS (Long et al., 2007; Luo et al., 2015; Xu et al., 2015; Sun et al., 2016). However, only a few genes underlying these QTLs have been identified. A deeper understanding of the genetic basis of these traits in rapeseed is essential for further improvement of yield.

Rapeseed was originated and domesticated in Europe, with cultivation spreading more recently to other continents. *B. napus* rapeseed varieties have been classified into two major growth types, winter (WOR) and spring oilseed rape (SOR), based on the vernalization requirement to flower. WORs require a long period of vernalization under low temperature and thus are hardy enough to endure the harsh, winter climates in Northern and Central Europe. SORs, which are grown in only during the spring and summer in Canada, northern Europe, northern China and the northern tier of the United States, do not require vernalization to flower. *B. napus* was first introduced to China in the 1930s and 1940s directly or indirectly from Europe (Liu, 1985), and primarily grown as a winter rotation crop following rice in the provinces along the Yangtze River, a subtropical region with a short and temperate winter. The earliest introduced varieties were late-flowering WORs, which postponed the following transplanting of rice. To meet the seasonal requirement for transplanting rice, rapeseed breeders hybridized the European WORs with the indigenous early-flowering *B. rapa* varieties. These endeavors created a new growth-type category of semi-winter oilseed rape (SWOR), which has a shorter life cycle and is specifically grown in China. SWORs are facultatively able to flower with or without vernalization, and thus can grow in both winter and spring environments.

In this study, we developed a rapeseed NAM population (BN-NAM) consisting of 2,425 F_6_ recombinant inbred lines (RILs) derived from crosses between the common parent Zhongshuang11 (ZS11) and 15 diverse SWOR and SOR founder lines. The objectives of this study were: (1) to develop a NAM population for the rapeseed research community, and especially for the SWOR research community; (2) to construct high-resolution linkage maps for individual RIL families and the whole NAM population; (3) to survey the genetic properties of the NAM population. The NAM population is expected to be a very useful resource for dissecting the genetic architecture of flowering, oil content, and yield-related traits in rapeseed, and especially valuable for the special growth-type SWOR.

## Materials and Methods

### Selection of a Diverse Set of Parents for BN-NAM Development

In our previous study, we collected 307 rapeseed inbred lines representing adaptations to the world major rapeseed production countries and evaluated the growth adaptability to the local climate in Wuhan, China. A panel of 192 inbred lines well-adapted to the semi-winter and spring growth environments was selected and genotyped using 451 single-locus SSR markers. Structure analysis divided the total panel into two groups, named P1 and P2. The P1 group was further subdivided into subgroup 1 (P1_1) and subgroup 2 (P1_2), and P2 into subgroup 1 (P2_1) and subgroup 2 (P2_2) (Xiao et al., 2012). Fifteen inbred lines representing the genetic diversity of SWOR and SOR were selected and used as the diversity donors based on clusters of genotypes as well as their phenotypic traits and growth types, with two to six lines from each main diversity group. In detail, six inbred lines (Zhongshuang2, 84001, Kangnongda, Gangan-F7, 352 and 264) were from P1_1, four (Baihua, Ribenyoucai, Zheyou7, Fuyou1) from P1_2, two from P2_1 (Quantum, CAo3Ho-4), and three from P2_2 (Bugle, DH4, DH5) (Xiao et al., 2012).

ZS11 was selected as the common male parent. It is an elite open-pollinated SWOR cultivar which is currently being widely cultivated in China, with many elite traits such as long siliques, more seeds per silique, large seeds, high oil content (49.6%), double-low quality, resistance to lodging and to *Sclerotinia sclerotiorum*. ZS11 has also been *de novo* sequenced and a high-quality reference genome was assembled (Sun et al., 2017). For simplicity, the common parent ZS11 was designated as D0 and the other 15 diverse inbred lines were designated as D1–D15 (Table 1). D0 served as the male parent and crossed to each diversity donor. More than one hundred F_2_ individuals were obtained from each cross. All F_2_ individuals were self-pollinated to F_6_ generation using the single-seed descent (SSD) method of bagged plants. The selection of parents (SWOR and SOR) ensured that all offspring lines could flower normally and seeds could be harvested in both semi-winter and spring growth environments. This ensured that we would obtain a NAM population consisting of RILs at their F_6_ generation within three and half years by shuttle breeding between semi-winter (Wuhan, Hubei Province) and spring (Hezheng, Gansu Province) environments. Winter type oilseed rapes (WORs) were excluded from the NAM population because they could not flower and set seeds in the spring growth environment. The 15 RIL families at their F_6_ generations were correspondingly designated as NAM01-15.

**Table 1:** Information for 16 parents of BN-NAM population.

No.^a^	Variety	Origin	Growth type^b^	Group	Sequence data (Gb)	Number of variants^c^
D0	ZS11	China	SWOR	G1	18.87	–
D1	ZS2	China	SWOR	G1	12.94	708,295
D2	Quantum	Canada	SOR	G3	12.70	853,956
D3	Bugle	Canada	SOR	G1	15.69	778,103
D4	DH4	China	SWOR	G4	11.56	1,029,575
D5	DH5	China	SWOR	G4	17.11	946,916
D6	84001	China	SWOR	G1	14.89	658,993
D7	Baihua	China	SWOR	G2	20.45	655,192
D8	CAo3Ho-4	Canada	SOR	G3	17.05	847,804
D9	Kangnongda	China	SWOR	G1	13.20	765,224
D10	Gangan-F7	China	SWOR	G1	15.02	561,374
D11	Ribenyoucai	Japan	SWOR	G2	16.39	664,644
D12	Zheyou7	China	SWOR	G2	14.50	686,758
D13	Fuyou1	China	SWOR	G2	17.84	570,790
D14	352	China	SWOR	G1	20.67	775,234
D15	264	China	SWOR	G2	13.01	831,025

*^*a*^D0 is the common male parent of RIL families, D1–15 are the female parents of RIL families.*

*^*b*^Semi-winter type oilseed rapes (SWOR) and spring type oilseed rapes (SOR).*

*^*c*^The number of variants compared to the common parent D0*.

### Whole Genome Resequencing and Genotyping of NAM Parents

Genomic DNA was extracted from young leaves using a cetyltrimethylammonium bromide (CTAB) method (Murray and Thompson, 1980). The quality of genomic DNA was checked on a 1% agarose gel and quantified using Qubit2.0 (Invitrogen). One μg genomic DNA was fragmented by sonication with a Biorupture (Diagenode; Liege, Belgium). DNA fragments ranging from 300 to 400 bp were purified using the Qiagen gel purification kit (Qiagen, Valencia, CA, United States). The sequencing library was constructed according to the Illumina manufacturer’s instructions and sequenced on the HiSeq2000 platform to produce paired-end 100 bp reads (PE100) (GenoSeq, Wuhan). The identification of single-nucleotide polymorphisms (SNPs) and insertions/deletions (INDELs) was done as previously described (Wang et al., 2018). The PE100 reads from individuals were first mapped to the reference genome sequence of ‘Darmor-*bzh*’ v4.1 (Chalhoub et al., 2014) by using the Burrows-Wheeler Alignment tool (BWA, version 0.7.0), with a maximum of three mismatches and one gap of 1 to 10 bp at each end. Then the alignment results were converted from SAM to BAM format using SAMtools (version 0.1.18). PCR duplications were identified and removed using the Picard package (version 1.91). Reads with gaps were realigned using the local realignment tool in Genome Analysis Toolkit (GATK, version 2.4). Calling and genotyping of SNPs and INDELs (1–10 bp) were performed using the UnifiedGenotyper module in GATK (version 2.4). To ensure the quality of markers used for further analysis, the identified variants were further filtered using the following criteria: (i) relative heterozygosity (HR) less than 0.2 (Wu et al., 2016); (ii) the percentage of missing genotype of one variant is less than 60%. The missing genotypes were imputed using Beagle (version 3.3.2).

### Construction of ddRAD Sequencing Library and Genotyping of BN-NAM

Genomic DNA was extracted from young leaves from a single plant of each RIL and quantified using Qubit2.0 (Invitrogen). All DNA samples were diluted to a similar concentration (approximately 50 ng/μl). A previously described ddRADseq technology was used to prepare genotyping-by-sequencing libraries (Chen et al., 2013). Briefly, 200 ng genomic DNA was double digested with *Mse*I and *Sac*I, and ligated with adaptors conferring different barcodes. Inserts with size ranging from 120 to 370 bp were recovered from 2% agarose gel and enriched by PCR using Illumina P5 and P7 primers. The PCR products were then purified with the Qiagen gel purification kit (Qiagen, Valencia, CA, United States) and sequenced on a HiSeq2000 platform to produce single-end 100 bp (SE100) reads from the *Sac*I side. Clean data were obtained by removing low-quality reads and parsed to individual RIL based on unique barcodes. SNP discovery and genotyping were conducted as previously described (Wu et al., 2016). Lines with ≤50% missing genotype data and ≤15% heterozygosity were kept for subsequent analyses. In addition, SNPs with ≤50% missing data, ≤20% heterozygosity and minor allele frequency (MAF) ≥ 15% were kept for genetic linkage maps construction.

### Construction of Individual Linkage Maps for Each RIL Family

For each RIL family, the filtered markers were first sorted into linkage group (LG) based on a LOD score ≥ 20 using a custom Perl script. To determine the order of markers within the same LG corresponding to a specific chromosome, the tag sequences of SNP markers were aligned to the *B. napus* reference genome of ‘Darmor-*bzh*’ v4.1 (Chalhoub et al., 2014). Next, a smoothing algorithm (Van Os et al., 2005) was used to filter out low-quality markers that resulted in too many double crossovers within a short distance on the reference genome sequence. Then, a HMM-based smoothing algorithm (Van Os et al., 2005) was used to impute the missing genotypes. Finally, the genetic distance and order for each marker were calculated based on the algorithm described in MSTMap (Wu et al., 2008).

### Construction of Joint Linkage Map for BN-NAM

A joint linkage map (JLM) was constructed for the BN-NAM population by integrating the 15 individual linkage maps. Firstly, the markers with inconsistent orders between the genetic and physical maps were deleted. Secondly, markers which were polymorphic in an individual RIL family were ordered according to their physical locations on the *B. napus* reference genome of ‘Darmor-*bzh*’ v4.1. Thirdly, individuals that were homozygous to any of the 15 donor parents were scored as “A”, individuals that were homozygous to the common parent (ZS11) were scored as “B”, and individuals that were heterozygous were scored as “H.” Markers monomorphic in a particular RIL family were scored as missing data “NA.” Fourthly, the missing data “NA” were imputed according to the genotypes of two adjacent flanking markers on the physical map. If the two adjacent flanking markers had the same genotype, the missing genotype “NA” was imputed to the genotype of the adjacent flanking markers. If the two adjacent flanking markers had different genotypes, the missing genotypes were imputed to the genotypes of the closest flanking marker based on their physical locations. Finally, the genetic distance for each marker was calculated based on the algorithm described in MSTMap (Wu et al., 2008).

### Construction of a Whole-Genome Variant Map by Projection

Sequence variations identified from the whole-genome resequencing data of the NAM parents were projected to the individual linkage map of each RIL family to construct a whole-genome variant map (WVM). The markers on individual linkage map of each RIL family were treated as skeleton markers. If a SNP or INDEL of parents was located between two skeleton markers, the genotype of the SNP or INDEL was imputed according to the genotypes of the two adjacent skeleton markers. If two adjacent skeleton markers had homozygous genotypes from the same parent, then the SNPs and INDELs of parents in the interval were projected to the individual linkage map with the genotypes of the two skeleton markers. If two adjacent skeleton markers had different genotypes derived from different parents, the SNPs and INDELs of parents in the interval were imputed to the genotypes of the closer skeleton marker based on physical location. Finally, the high-density maps generated by each RIL family were combined into a WVM of BN-NAM.

### Population Structure of BN-NAM Population

Markers located in the intergenic regions usually experience weak selective pressure, and so more reliably reflect population structure and demography (Lin et al., 2014). To reflect the actual population structure of the NAM population, a subset of 6,112 intergenic markers evenly distributed across the genome were selected with a distance between two adjacent markers greater than 100 Kb on the WVM. These were used to assess the population structure of BN-NAM. Population structure of the BN-NAM was analyzed by STRUCTURE 2.3.4 software (Evanno et al., 2005). The number of ancient clusters *K* was set from 1 to 10, and the analysis was repeated five times. We used a model without admixture and correlated allele frequencies. A burn-in period of 50,000 and Monte Carlo Markov Chain replicates of 100,000 were used. The natural logarithms of probability data [LnP(K)] and the *ad hoc* statistic Δ*K* were calculated using STRUCTURE HARVESTER: an online program for visualizing STRUCTURE output that implements the Evanno method^2^ (Earl and vonHoldt, 2012). The kinship (*K*) matrix was estimated using the same subset of 6,112 markers by SPAGeDi (version 1.4b) (Hardy and Vekemans, 2002). Principal component analysis (PCA) was based on a subset of 79,359 evenly distributed markers located in intergenic regions selected with a distance greater than 5 Kb between adjacent markers. PCA was performed with the smartpca program of EIGENSOFT (version 5.0.1) software (Price et al., 2006), and the first two eigenvectors were plotted in two dimensions with a custom R script.

### Linkage Disequilibrium

Based on 82,998 evenly distributed markers (the distance to adjacent variants greater than 5 Kb) on the WVM, the pattern of linkage disequilibrium (LD) across the *Brassica* A and C sub-genomes was estimated based on the parameter *r*^2^ of all pairwise markers with unique physical positions using the software TASSEL version 5 (Bradbury et al., 2007). LD decay of the BN-NAM population was estimated based on the physical distance of all pairwise markers. The distance of LD decay for each chromosome is defined as the *r*^2^ value decreased to half of the maximum (Dell’Acqua et al., 2015). The local LD decay across chromosome is depicted as the average *r*^2^ of all marker pairs within an 800 Kb (±400 Kb) chromosomal region. This length (800 Kb) of chromosome region was chosen as 800 Kb, which corresponds to the intermediate halving distance of LD decay according to LD analysis of the chromosomes.

### Analysis of Segregation Distortion

Allele frequency was calculated for each locus on individual linkage maps. For each locus in a RIL population, the expected ratio of the two alleles is 1:1. A chi-square test (χ^2^) was performed to determine whether the allele frequency significantly differed from the expectation of 1:1 (*P* < 0.05, df = 1). A stretch of two or more closely linked markers (adjacent markers ≤10 cM) exhibiting segregation distortion at a more stringent threshold (*P* < 0.001) level was defined as a segregation distortion region (SDR).

## Results

### Development of the BN-NAM Population

In order to develop a NAM population for linkage and association studies of complex traits in oilseed rape, especially in SWORs, a subset of 15 inbred lines (D1–15) were selected to represent most of the genetic diversity of the SWOR and SOR germplasm from an association panel of 192 inbred lines (Table 1). These 15 inbred lines were crossed with ZS11 (D0) for the creation of RIL families by single-seed descent (SSD) (Supplementary Figure S1B). The BN-NAM population contained 15 RIL families and a total of 2,425 RILs, with between 117 and 213 lines per RIL family.

To further assess the whole genome variation of the NAM parents, we re-sequenced the 15 diversity donors and the common parent. A total of 252 Gb sequence data were obtained for the 16 NAM parents, with a depth of genome coverage ranging from 10× to 18× (Table 1). A total of 4,444,309 variants, including 3,885,328 SNPs and 558,981 INDELs (1–10 bp), were identified between the diversity donors and the common parent (D0) (Supplementary Table S1). A neighbor-joining tree was constructed from the whole-genome variation dataset (Supplementary Figure S1A). The 16 NAM parent lines were divided into four groups, which is similar to a previous outcome (Xiao et al., 2012). Seven lines (D0, D1, D3, D6, D9, D10, and D14) formed group 1 (G1), five (D7, D11, D12, D13, and D15) formed G2, two (D2 and D8) formed G3, and two (D4 and D5) formed G4 (Supplementary Figure S1A).

### Individual Linkage Maps for RIL Families

All 2,425 RILs and the 16 parents were genotyped by sequencing. A total of 4.40 × 10^9^ SE100 reads containing barcodes and partial recognition sequences were generated, and the mean number of reads per RIL of 1.80 million. After filtering, 141,687 high-quality SNP markers were retained for the 15 RIL families, with between 6,096 and 13,093 markers per RIL family. There were a total of 2,141 RILs were retained for genetic linkage map construction after elimination of 284 RILs. Individual linkage maps were constructed for the 15 RIL families. SNP markers on individual linkage maps were aligned to the *B. napus* reference genome, which allowed us to detect the regions that with inconsistent markers orders between the genetic and physical location (Supplementary Figure S2). On average, 127 conflicting regions were detected for each RIL family and the total length of these regions was 55 Mb. Based on the distribution of conflicting regions on the genomic chromosomes, we found that the number of conflicting regions on the A sub-genome is greater than that of the C sub-genome, and the large-length conflicting regions mostly occurred in the pericentromeric regions. For example, in the pericentromeric region on chromosome A6, a conflicting region (9.6–15.8 Mb) was detected in all RIL families except the NAM05 (Supplementary Figure S2). In order to integrate genetic linkage maps for individual RIL families and analyze population properties on the same scale, we removed the markers that with inconsistent orders between the genetic and physical location. Individual linkage maps had 4,079 to 9,375 SNP markers and a total of 94,701 redundant SNP markers across the 15 linkage maps (Table 2). It is worth pointing out that, on the linkage map of the NAM14 family, two homoeologous chromosomes, A02 and C02, had no markers due either to homoeologous non-reciprocal transposition (HNRT) or homoeologous exchange (HE) occurring between A02 and C02 of D14 (352) (Chen et al., 2017). The individual linkage maps ranged in length from 1,564.7 to 2,446.2 centiMorgans (cM), with an average length of 1,989.7 cM. The average genetic distance between two adjacent markers was 0.32 cM, with a range of 0.20–0.49 cM on individual linkage maps (Table 2).

**Table 2:** Summary of linkage maps for 15 RIL families.

Families	Number of lines	Map length (cM)	Markers mapped	Average genetic distance (cM)^a^	Recombination rate (cM/Mb)
NAM01	152	2031.4	6,912	0.29	3.15
NAM02	145	2038.7	7,892	0.26	3.16
NAM03	114	2446.2	6,621	0.37	3.79
NAM04	134	1962.3	8,033	0.24	3.04
NAM05	173	1882.2	9,375	0.20	2.92
NAM06	148	1941.3	5,344	0.36	3.01
NAM07	183	2322.1	5,724	0.41	3.60
NAM08	144	1992	7,659	0.26	3.09
NAM09	138	1990.9	6,112	0.33	3.08
NAM10	132	1967.5	6,116	0.32	3.05
NAM11	138	1564.7	4,079	0.38	2.42
NAM12	177	1741.4	5,538	0.31	2.70
NAM13	142	1876.3	4,787	0.39	2.91
NAM14	97	2166.9	4,405	0.49	3.36
NAM15	124	1922.2	6,104	0.31	2.98
Total	2,141	29,846.1	94,701	0.32	3.08

*^*a*^Average genetic distance between successive markers*.

Co-segregating markers with no detectable recombination within a block on an individual RIL linkage map formed a marker bin. The number of bins for individual RIL linkage maps ranged from 1,308 in NAM11 to 2,805 in NAM05, with an average of 1,834 bins across all RIL families. The number of bins on each chromosome for the 15 RIL families is provided in Supplementary Table S2.

### Joint Linkage Map and Whole-Genome Variant Map of BN-NAM

Among the 94,701 SNP markers on the 15 linkage maps, 13,374 were unique to single maps, whilst the remaining 81,327 markers were shared by two or more linkage maps. For instance, 6,414, 3,425 and 2,458 markers are shared in common by two, three, and four linkage maps, respectively, and 1,129 markers are shared in common by more than 10 linkage maps. By aligning the common markers among the 15 linkage maps, we constructed a JLM for the BN-NAM, which contained 30,209 non-redundant markers. These markers formed 10,182 bins, which are evenly distributed across the 19 LGs, ranging from 368 bins on chromosome A08 to 901 bins on chromosome C03 (Table 3). The JLM has a total genetic distance of 1611.18 cM, with an average chromosome length of 87.98 cM, ranging from 53.36 cM on chromosome A04 to 127.61 cM on chromosome C03. The average genetic distance between two adjacent bins was 0.158 cM, with a range of 0.116 cM on chromosome C02 to 0.233 cM on chromosome C06. The average bin length corresponded to a physical distance of 28 Kb, and the average physical distance between bins across the genome was 66 Kb. These values were calculated based on the physical locations of markers within and between bins in relation to the reference pseudochromosome genome sequences of ‘Darmor-*bzh*’ v4.1 (Table 3).

**Table 3:** Summary of joint linkage map for BN-NAM population.

LG^a^	Number of bins^b^	Chr. length (cM)	Average genetic distance (cM)^c^	Average intra-bin distance (Kb)	Average inter-bin length (Kb)
A01	515	107.31	0.208	18	45
A02	442	99.69	0.226	22	56
A03	740	92.63	0.125	15	40
A04	422	53.36	0.126	17	45
A05	507	88.23	0.174	17	88
A06	645	97.36	0.151	15	38
A07	552	94.62	0.171	14	43
A08	368	63.00	0.171	21	51
A09	674	112.80	0.167	18	50
A10	507	61.57	0.121	13	34
C01	455	71.34	0.157	46	85
C02	620	71.89	0.116	34	74
C03	901	127.61	0.142	30	67
C04	514	104.87	0.204	41	95
C05	416	95.85	0.230	44	104
C06	409	94.83	0.232	48	114
C07	490	99.39	0.203	41	91
C08	510	74.82	0.147	38	74
C09	495	60.35	0.122	46	98
Total	10,182	1611.18	0.158	28	66

*^*a*^Linkage groups.*

*^*b*^The number of bins of each linkage group.*

*^*c*^Average genetic distance between successive bins*.

To build a whole-genome variant map (WVM) for the BN-NAM population, all the 4,444,309 polymorphic markers between D0 and the donor parents were projected to the individual linkage maps as described (Xiao et al., 2016). The distribution of SNPs and INDELs varied dramatically between the A and C sub-genomes (Supplementary Table S1). The density of SNPs in the A sub-genome was 8.3/kb and of INDELs 1.4/kb, about twice of that in the C sub-genome (4.7/kb SNPs and 0.6/kb for INDELs) (Supplementary Table S1).

### Population Structure and LD Analyses of BN-NAM

**FIGURE 1:**
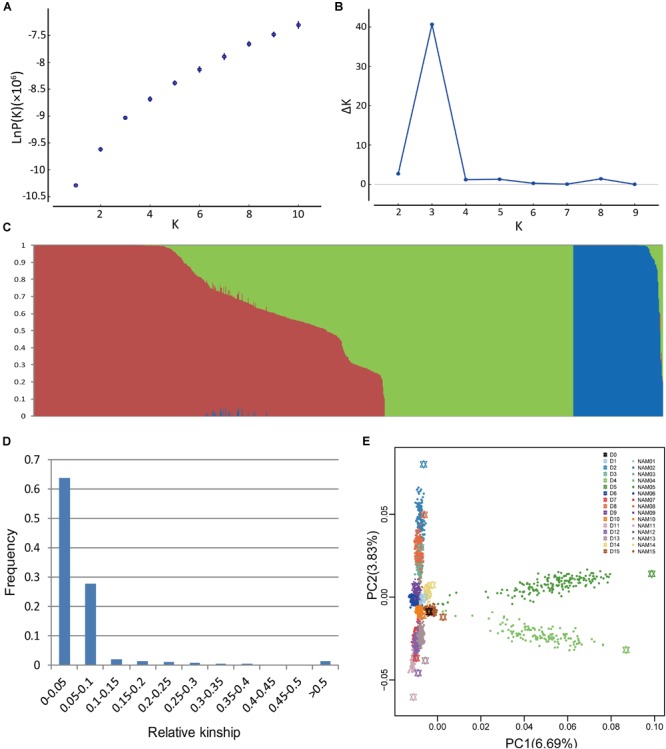
Analyses of population structure and relative kinship coefficient for the BN-NAM. **(A)** Estimated LnP(D) of possible clusters (K) from 1 to 10. **(B)** Delta K based on rate of change of LnP(D). **(C)** Population structure of the BN-NAM population revealed by STRUCTURE. When *k* (the number of subpopulations) is at 3, BN-NAM was classified into three groups. **(D)** Distribution of pairwise relative kinship estimates between all lines of BN-NAM. **(E)** Principal component analysis (PCA) plots of the first two components of BN-NAM RILs. Different color points represent different families. The black hexagon in the middle is the common parent “ZS11” and the other color hexagons represent the other donor parents (D1–15). Each solid dot represents a recombinant inbred line (RIL). The same color hexagon and dots indicates the donor parent and its corresponding RIL family, respectively.

Population structure is a possible factor to cause spurious associations in GWAS (Flint-Garcia et al., 2005). NAM gives rise to a particular population structure due to the inherent genetic architecture of the cross design, which we analyzed in BN-NAM using the STRUCTURE software. The whole BN-NAM population could be divided into three groups (Figures 1A–C).

Principal component analysis also showed that all the offspring of BN-NAM were grouped into three main clades (clades 1, 2, and 3) surrounding the common parent (D0), and which corresponded to the Groups I, II and III revealed by STRUCTURE analysis (Figure 1E). The top two principal components, PCA1 and PCA2, individually explained 6.69% and 3.83% of the genetic variance, and jointly explained 10.52% genetic variance, suggesting that the BN-NAM population had weak stratification. Moreover, 63.7% of the kinship coefficients between lines of BN-NAM were between 0 and 0.05 (Figure 1D), indicating a very weak relationship between most lines.

**FIGURE 2:**
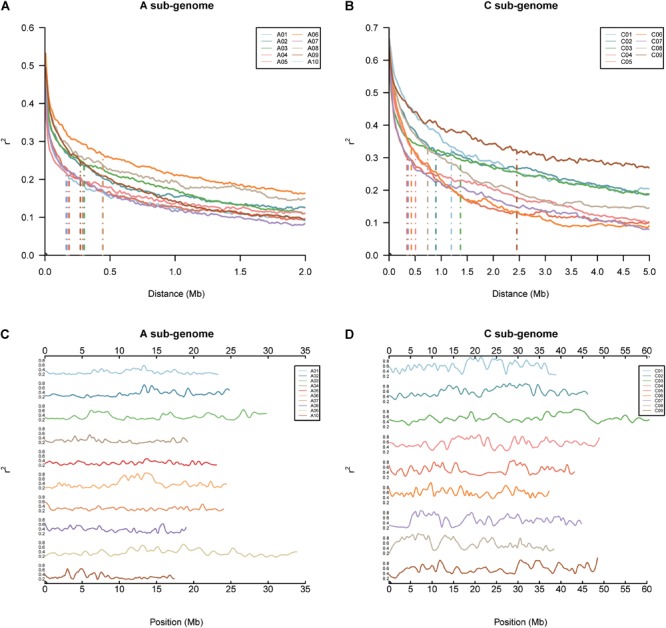
Genomic linkage disequilibrium (LD) analyses in the BN-NAM. **(A,B)** LD decay in different chromosomes in the A and C sub-genome, respectively. **(C,D)** Distribution of local LD along chromosomes in the A and C sub-genome, respectively. Local LD pattern was calculated based on the average *r*^2^ value of each marker within an 800 Kb (±400 Kb) window.

The pattern of LD in the BN-NAM population was analyzed by calculating *r*^2^ values of all pairwise markers within each chromosome. The LD decay distance (*r*^2^ decay to half of the maximum) across the 19 chromosomes in whole genome varied between 170 and 2,400 Kb. The mean LD decay distance in the A sub-genome is 280 Kb, varying from 170 to 450 Kb, and while the mean LD decay distance in the C sub-genome is 950 Kb, ranging from 360 to 2,400 Kb. LD in the A sub-genome decays much faster than in the C sub-genome (Figures 2A,B). LD decay is also variable in different genomic regions (Hyten et al., 2007). Large LD blocks were consistently located in the centromere regions (Mason et al., 2016). The local LD pattern in the BN-NAM genomes was calculated based on the average *r*^2^ value of each marker within a ± 400 Kb window. In the BN-NAM genomes, the average *r*^2^ value was 0.60 for the markers within ±1 Mb of the pericentromeric regions and averaged 0.36 for the markers outside the centromeres, a 67% increase in pericentromeric regions. We found higher average *r*^2^ value of markers in pericentromeric regions on all 19 chromosomes (*P* = 6.17 × 10^-81^) (Figures 2C,D), which suggests that the pericentromeric regions represent large LD blocks in the genome, especially in the C sub-genome (Figure 2). The C sub-genome has many more regions with larger LD blocks than the A sub-genome.

### Estimation of Recombination Rate and Recombination Events

Recombination events (REs) were measured in each RIL by counting breakpoints in markers ordered according to their map position on each chromosome, where stretches of SNP alleles from one parent switch to the variant of the other parent. We counted REs on each chromosome based on the individual genetic linkage map of each RIL family. The number of REs varied from 4,109 in NAM14 to 8,603 in NAM07, with a total of 88,386 unique REs identified in the BN-NAM population. The mean number of REs per line varied from 32.6 in NAM11 to 50.7 in NAM03, with average REs of 41.4 per line in the BN-NAM population (Supplementary Figure S3). REs represent chiasmata that form during prophase I of meiosis. The average number of REs per line per chromosome varied from 1.51 in chromosome C04 to 3.44 in chromosome C03, with an average REs of 2.17. The average number of REs per line per chromosome was positively correlated with chromosome physical length (*r* = 0.57, *P* = 0.01), indicating that longer chromosomes accumulate more REs. In the A sub-genome these varied from 3,229 in chromosome A04 to 6,110 in chromosome A09, with a total of 44,430 unique REs in the A sub-genome, In the C sub-genome, REs varied from 3,710 in chromosome C09 to 7,374 in chromosome C03, with a total of 43,956 unique REs in the C sub-genome (Supplementary Table S3). But, REs in unit length (/Mb) of A sub-genome was 182, a nearly two-fold difference of C sub-genome (108/Mb) (Supplementary Table S3).

**FIGURE 3:**
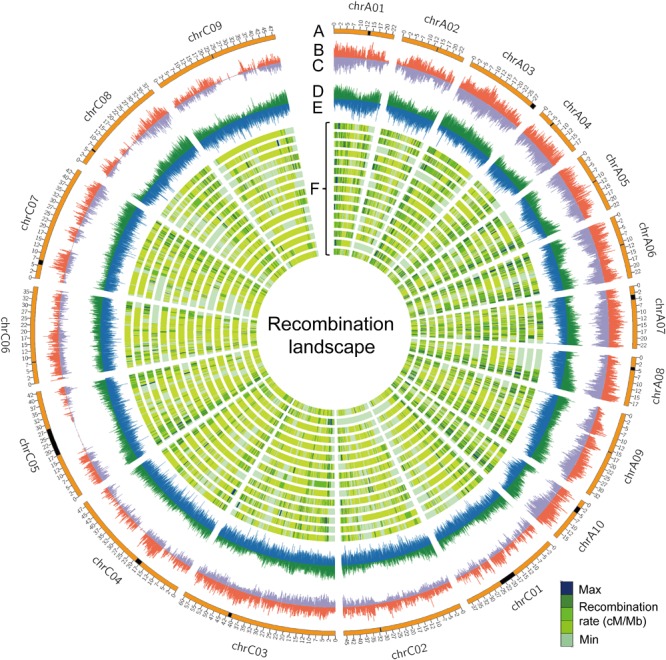
The recombination landscape in all RIL families. **(A)** Chromosomes of the *B. napus* genome, where the black segments represent the pericentromeric regions. **(B,C)** Indicate the whole-genome SNP and INDEL densities along chromosomes, respectively. The sliding window was set to 100 Kb with the densities of genes **(D)** and transposable elements (TEs) **(E)** indicated along chromosomes of the reference genome ‘Darmor-*bzh*’ v4.1. **(F)** The genome-wide landscape of recombination (cM/Mb) is defined using a sliding window of 100 Kb across 15 RIL families. Lanes from outer to inner are NAM01 to NAM15.

The rate of recombination (cross-over frequency), which can fragment haplotypes by re-assorting chromosome segments, is one of the main factors that determine the distance of LD decay. The rate of recombination varies along chromosomes and is reflected in variation of genetic distance per megabase pairs (cM/Mb) (Dumont et al., 2011). We surveyed the genome-wide recombination rate (GWRR), measured as the ratio of the genetic distance in centiMorgans (cM) to the physical genome size in megabase pairs (cM/Mb), in different RIL families. The GWRR ranged from 2.42 cM/Mb in NAM11 to 3.79 cM/Mb in NAM03, with an average of 3.08 ± 0.32 cM/Mb in the BN-NAM population (Table 2). To depict the distribution pattern of recombination rate along chromosomes, we calculated the genetic distance/Mb with a sliding window to reflect the locus recombination ratio (LRR) (Pan et al., 2016). From the individual linkage map of each RIL family, we used markers with consistent orders in both the genetic and physical maps and summarized the recombination as cM/Mb using a 100-Kb window size. The LRR varied from 0 (no recombination events) to 25.8 cM/Mb along the *B. napus* chromosomes (Figure 3F). The average LRR was 1.19 within ±1.0 Mb in the centromere regions, while it was 3.40 outside the centromere regions, which suggested that the interstitial and distal regions of chromosomes had higher LRR (*P* = 2.3 × 10^-268^) and represent recombination hot spots. In addition, the densities of genes and transposable elements (TEs) along chromosomes were defined using a sliding window of 100 Kb, as were SNP and INDEL densities of WGM and average *r*^2^ values. We calculated the correlation between the LRRs and the densities of genes, TEs, SNP and INDELs and LD (average *r*^2^ values) along chromosomes. We found that the distribution of LRRs along chromosomes is positively correlated with the densities of genes (*r* = 0.57, *P* = 0), SNPs (*r* = 0.32, *P* = 1.25 × 10^-150^) and INDELs (*r* = 0.52, *P* = 0), but negatively correlated with the density of TEs (*r* = -0.28, *P* = 6.17 × 10^-115^) and LD (*r* = -0.48, *P* = 0) (Figure 3 and Supplementary Figure S4). These results indicate that recombination is context dependent, and more likely to occur in regions having a greater density of genes, similar to the findings in maize (Pan et al., 2016).

### Segregation Distortion Analysis

**FIGURE 4:**
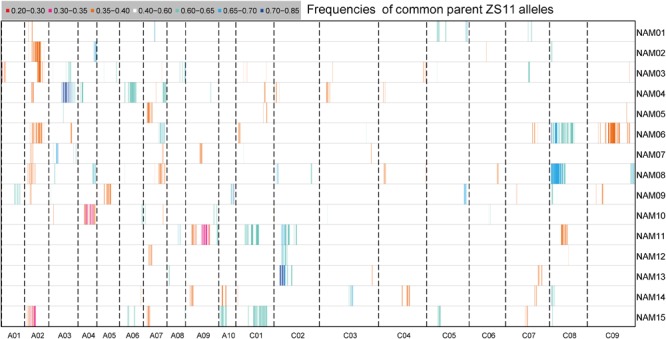
The distribution of segregation distortions along chromosomes in the RIL families. The horizontal lines represent the 15 RIL families (NAM01–15) and the vertical columns represent the 19 chromosomes (A01–10 and C01–09). The frequency of ZS11 alleles for an interval family is indicated by the color scale.

Segregation distortion (SD) is a common phenomenon observed in animal and plant segregating populations where zygote loci deviate from the expected Mendelian ratios of gametic alleles (Matsubara et al., 2011). We surveyed the segregation ratio of all markers on the individual linkage map of each RIL family. Within the 15 RIL families, a total of 3,231 (11.75%) markers exhibited segregation distortion, ranging from 4.05% in the NAM14 to 17.88% in the NAM08 (*P* < 0.05) (Table 4 and Figure 4). A total of 2,284 (70.69%) markers distorted to ZS11, whereas 947 (29.31%) markers distorted to the donor parents (*P* < 0.05) (Table 4). This suggests that the alleles from the common male pollen parent have an advantage over the alleles from the female donor parents. For most RIL families, markers on the same chromosomes tend to be over-represented (distorted segregation) in the same direction. For a relatively small number of chromosomes some markers were over-represented by alleles from one parent, whilst the remaining markers were distorted toward alleles of the other parent.

**Table 4:** Number and frequency of bins displaying segregation distortion in the 15 RIL families, *P* < 0.05.

Families	Number of bins	Number of SD bins (%)^a^	Number of bins distorted to D0 (%)^b^	Number of bins distorted to D1–15 (%)^c^
NAM01	1,768	193 (10.92)	158 (81.87)	35 (18.13)
NAM02	2,057	223 (10.84)	142 (63.68)	81 (36.32)
NAM03	1,857	167 (8.99)	130 (77.84)	37 (22.16)
NAM04	2,227	265 (11.9)	198 (74.72)	67 (25.28)
NAM05	2,805	321 (11.44)	275 (85.67)	46 (14.33)
NAM06	1,700	270 (15.88)	155 (57.41)	115 (42.59)
NAM07	1,988	212 (10.66)	133 (62.74)	79 (37.26)
NAM08	2,097	375 (17.88)	223 (59.47)	152 (40.53)
NAM09	1,666	185 (11.10)	140 (75.68)	45 (24.32)
NAM10	1,723	262 (15.21)	170 (64.89)	92 (35.11)
NAM11	1,308	186 (14.22)	127 (68.28)	59 (31.72)
NAM12	1,806	99 (5.48)	64 (64.65)	35 (35.35)
NAM13	1,481	169 (11.41)	139 (82.25)	30 (17.75)
NAM14	1,310	53 (4.05)	40 (75.47)	13 (24.53)
NAM15	1,713	251 (14.65)	190 (75.70)	61 (24.30)
Total	27,506	3,231 (11.75)	2,284 (70.69)	947 (29.31)

*^*a*^Number and frequency of segregation distortion bins of each RIL family.*

*^*b*^Number and frequency of segregation distortion bins of each RIL family distorted to common parent (D0).*

*^*c*^Number and frequency of segregation distortion bins of each RIL family distorted to other donor parents (D1–15)*.

Clusters of two or more adjacent distorted markers were regarded as segregation distorted regions (SDR). In total, 44 SDRs were found within 15 RIL families. If SDRs in different RIL families had overlapping intervals of confidence, they were considered to be the same and were integrated. Twenty-nine common SDRs were obtained, with no SDR shared by all families. Eight SDRs were shared in at least two RIL families. The SDRs on chromosome C02 and C08 each were shared by four RIL families (Supplementary Table S4).

### Residual Heterozygous Lines

Residual heterozygous lines (RHLs) are useful genetic resources for fine mapping of QTL, with the powerful attribute that self-pollination can generate near-isogenic lines for heterozygous chromosome regions. At the F_6_ generation of a RIL population, it is expected that the rate of heterozygous genotypes (Aa) is 3.13%. We calculated the residual heterozygosity for each RIL family and found that this varied from 1.49% in NAM01 family to 3.34% in the NAM05 family, with a mean level of 2.67% in the whole BN-NAM population (Supplementary Figures S5, S6). When we interrogated residual heterozygosity across the 19 chromosomes of each RIL family, we found that coverage of all chromosomes was represented along nearly the full length for each RIL family. The total length of heterozygous chromosome segments in all RILs was 37.3 Gb, which is equivalent to a genome coverage depth 33×. The heterozygous regions were evenly distributed over the whole genome and represents a valuable genetic resource for cloning and modification of QTL genes controlling important traits.

## Discussion

In rapeseed, the genetic architecture of complex traits has traditionally been dissected by QTL mapping using segregating populations derived from biparental crosses (Long et al., 2007), and more recently by GWAS using landraces, cultivars and breeding lines (Luo et al., 2015). Recently, the NAM design, which simultaneously exploits the advantages of both joint linkage analysis and association mapping, has been implemented in maize to dissect the genetic basis of complex quantitative traits (McMullen et al., 2009). NAM makes use of both historic and recent recombination events, and has the advantages of low marker density requirements, high allele richness, high mapping resolution, and high statistical power. At the same time it carries none of the disadvantages of either linkage analysis or association mapping. These advantages of NAM population design in dissecting the genetic basis of complex traits have attracted researchers and breeders to develop NAM populations in many other crops (Maurer et al., 2015; Bajgain et al., 2016; Bouchet et al., 2017; Fragoso et al., 2017; Song et al., 2017). In this study, we developed a NAM population consisting of 15 RIL families with 2,425 immortal genotypes derived from the crosses between the common parent ZS11 and 15 diverse founder lines. The 16 founders were selected to represent most of the genetic diversity of SWORs and SORs well-adapted to the winter growth environments along the Yangtze River and the spring growth environments in northern China. The NAM population was developed by shuttle breeding between winter and spring environments, and thus all the RILs could flower and set seeds in both of these growth environments. Therefore the BN-NAM population is especially valuable for dissecting the genetic architecture of complex traits in SWORs, a novel growth type of rapeseed specific to China, and SORs.

The major motivation to develop the NAM population was to dissect the genetic basis of complex traits by QTL linkage mapping or GWAS. GBS is an efficient and economical method, especially for genotyping RIL populations (Li et al., 2016). We thus constructed high-density genetic linkage maps for individual RIL families using the cost-effective GBS method, with each map consisting of 4,079–9,375 SNP markers (Table 2). The high-density linkage maps laid the foundation for QTL mapping of complex traits in multiple populations. Based on common markers shared by multiple linkage maps, we integrated the 15 linkage maps into a joint linkage map which contained 30,209 unique markers. The JLM consisted of 10,182 bins across 2,141 lines (Table 3), which is much higher than that in any previous studies in rapeseed, and thus should be powerful for dissecting the genetic basis of complex traits by joint linkage analysis. It is worth to point out that, when constructing the JLM, the markers that with inconsistent orders between the genetic and physical locations were removed, and translocations and inversions in single linkage map were omitted, which may have minor effects on mapping accuracy of QTLs located in these regions. Re-sequencing of the 16 founders identified 4,444,309 variations between the common parent ZS11 and the 15 donor parents. These whole-genome variations were projected onto the individual linkage maps to generate an ultra-dense whole-genome variation map (Figures 3B,C and Supplementary Table S1), which contained the historical recombination events of parents and the contemporary recombination events of all lines. The value of the BN-NAM population for GWAS of complex traits is to be expected.

Although the NAM design is expected to have a significant effect on population structure (Rafalski, 2010), few studies have characterized this structure. The BN-NAM population was clearly clustered into three groups with weak stratification (Figure 1C), compared to the lack of any significant stratification in MAGIC populations (Dell’Acqua et al., 2015) and significant stratification displayed in ROAM populations (Xiao et al., 2016). This suggests that the genetic properties of the BN-NAM population are quite distinct. The full-sib RILs of BN-NAM were clustered in small clusters according to their donor female parents, while the half-sib RILs were more dispersed around the common male parent. If the female parents originated from the same group (Supplementary Figure S1A and Table 1), their offspring was assigned to the same group as expected (Figure 1E), which is similar to the findings in the sorghum NAM population (Bouchet et al., 2017). These results suggested that the BN-NAM population retains genetic diversity but has a low population structure.

The concept of LD describes the non-random association of alleles at two or more loci caused by genetic linkage. Rapeseed is a relatively young allotetraploid species, which formed just 7,500 years ago and has only experienced about 400 years of domestication and cultivation (Chalhoub et al., 2014). Previous estimates of the distance of LD decay in rapeseed range from 0.25 to 2.5 Mb (Qian et al., 2014) or from 0.4 to 2.1 Mb (Wu et al., 2016). We observed that LD decay in the BN-NAM ranged from 170 to 2,400 Kb across the whole genome, which is in a similar range and is comparable to other polyploid crop species (Zhou et al., 2015; Huang et al., 2017). Moreover, we detected local LD in the centromere regions, especially on the C sub-genome chromosomes, which extended far longer than other regions (Figures 2C,D). LD decay in the C sub-genome is much slower than that in the A sub-genome (Figures 2A,B). Recombination is the main phenomenon that weakens intra-chromosomal LD (Flint-Garcia et al., 2003). Indeed, the landscape of genome-wide recombination within the BN-NAM population indicated that there are differences between the A and C sub-genomes (Figure 3F and Supplementary Table S3). Recombination also determines the resolution of QTL mapping, as well as the probability that favorable alleles combine during human and natural selection of plants and animals (Gaut et al., 2007). In the BN-NAM population, the total number of REs was 88,542 and the average number of REs was 41 per RIL, which is comparable to that in the NAM populations of other crops (McMullen et al., 2009; Pan et al., 2016; Song et al., 2017). We thus expect that the BN-NAM population would be as valuable as the maize NAM population in dissecting the genetic architecture of complex traits in rapeseed.

## Ethics Statement

The authors declare that the experiments comply with the current laws of the country in which they were performed.

## Author Contributions

JH, JY, ML, ZW, and QZ performed the experiments. JH, KL, and GK wrote the manuscript. JH, CG, BW, YX, and HL contributed to data analysis. KL conceived and supervised the study. All authors read and approved the final manuscript.

## Conflict of Interest Statement

The authors declare that the research was conducted in the absence of any commercial or financial relationships that could be construed as a potential conflict of interest. The reviewer FX declared a shared affiliation, with no collaboration, with several of the authors JH, CG, BW, JY, ML, ZW, YX, QZ, HL, and KL to the handling Editor at the time of review.

## Footnotes

1 https://apps.fas.usda.gov/psdonline/psdReport.aspx

2 http://taylor0.biology.ucla.edu/structureHarvester/

## Supplementary Material

FIGURE S1 The relationship of all founder lines and the scheme for development of BN-NAM population. **(A)** The neighbor-joining tree for 16 parents (P0–15) of BN-NAM. **(B)** Strategy for development of the 2,425 recombinant inbred lines (RILs) for BN-NAM.

FIGURE S2 The distribution of non-colinear regions between physical position and genetic position in genome. The left non-colinear regions of each chromosome in the figure are detected in at least seven RIL families. The dark spots on chromosomes represent centromeres.

FIGURE S3 Fig Boxplot of recombination events for each line in individual RIL family.

FIGURE S4 Correlation between recombination rate and its relating factors. **(A–E)** Correlation between recombination rate and the densities of genes, TEs, SNP, INDEL, and LD using a sliding window of 100 Kb, respectively. The recombination rate is calculated as the average of recombination rate of all RIL families in Figure 3F. The densities of genes, TEs, SNP and INDEL come from the data of Figures 3B,D.

FIGURE S5 Boxplot of residual heterozygosity for each line in individual RIL family.

FIGURE S6 Residual heterozygous regions along the 19 chromosomes in all lines of the BN-NAM population.

TABLE S1 The number and density of markers on the whole-genome variant map.

TABLE S2 The number of bins on bin maps for 15 RIL families.

TABLE S3 The number and density of recombination events along chromosomes of the BN-NAM population.

TABLE S4 Common segregation distortion region (SDR) identified in 15 RIL families, *P* < 0.001.
