# Supplementary figures and images for: Genetic Properties of a Nested Association Mapping Population Constructed With Semi-Winter and Spring Oilseed Rapes
Source: Front Plant Sci. 2018 Nov 26;9:1740. doi: 10.3389/fpls.2018.01740 (PMC6275288; doi:10.3389/fpls.2018.01740)

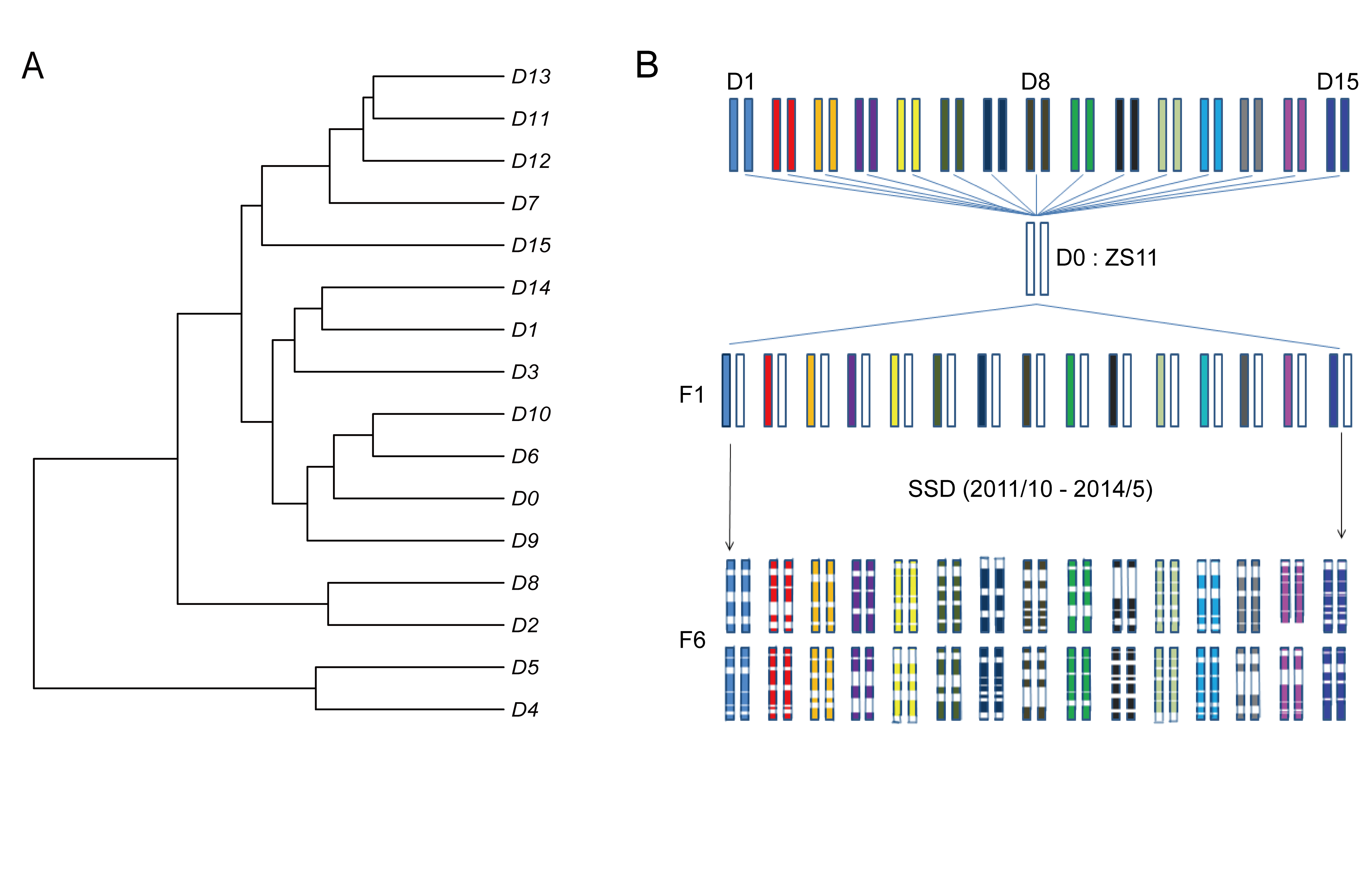

Supplement: FIGURE S1 — The relationship of all founder lines and the scheme for development of BN-NAM population. (A) The neighbor-joining tree for 16 parents (P0–15) of BN-NAM. (B) Strategy for development of the 2,425 recombinant inbred lines (RILs) for BN-NAM. [file Image_1.TIF]

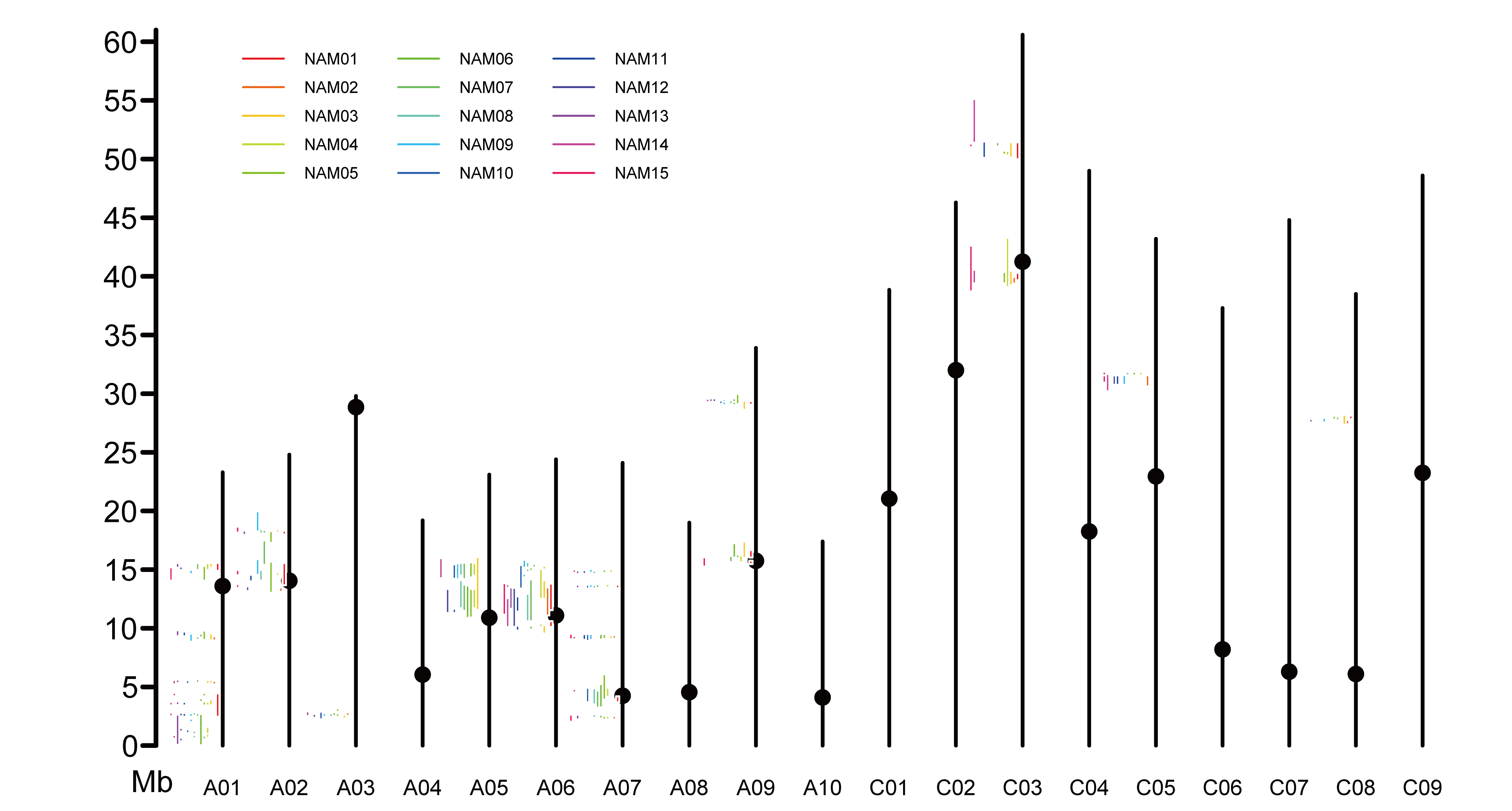

Supplement: FIGURE S2 — The distribution of non-colinear regions between physical position and genetic position in genome. The left non-colinear regions of each chromosome in the figure are detected in at least seven RIL families. The dark spots on chromosomes represent centromeres. [file Image_2.TIF]

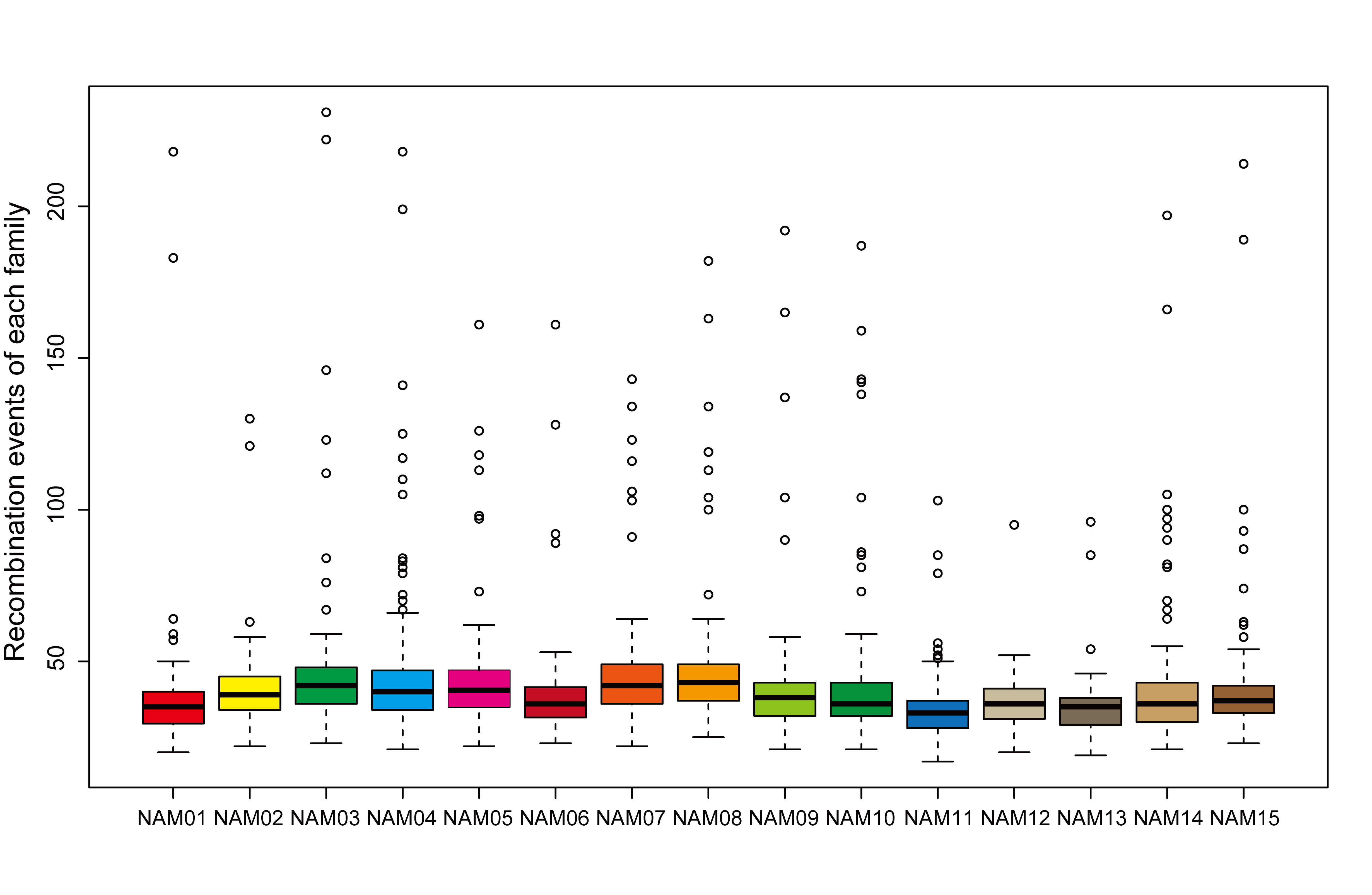

Supplement: FIGURE S3 — Fig Boxplot of recombination events for each line in individual RIL family. [file Image_3.TIF]

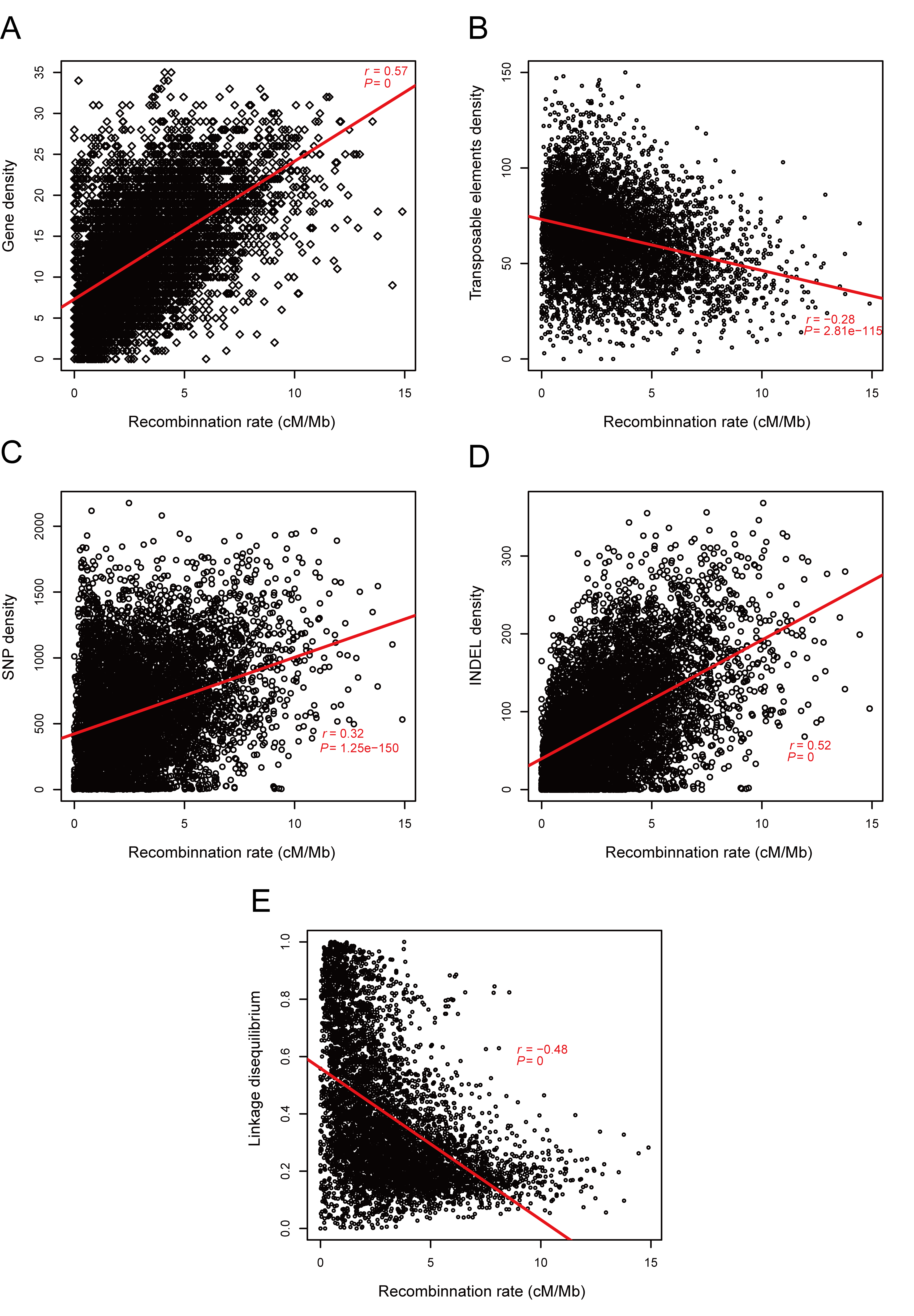

Supplement: FIGURE S4 — Correlation between recombination rate and its relating factors. (A–E) Correlation between recombination rate and the densities of genes, TEs, SNP, INDEL, and LD using a sliding window of 100 Kb, respectively. The recombination rate is calculated as the average of recombination rate of all RIL families in Figure 3F. The densities of genes, TEs, SNP and INDEL come from the data of Figures 3B,D. [file Image_4.TIF]

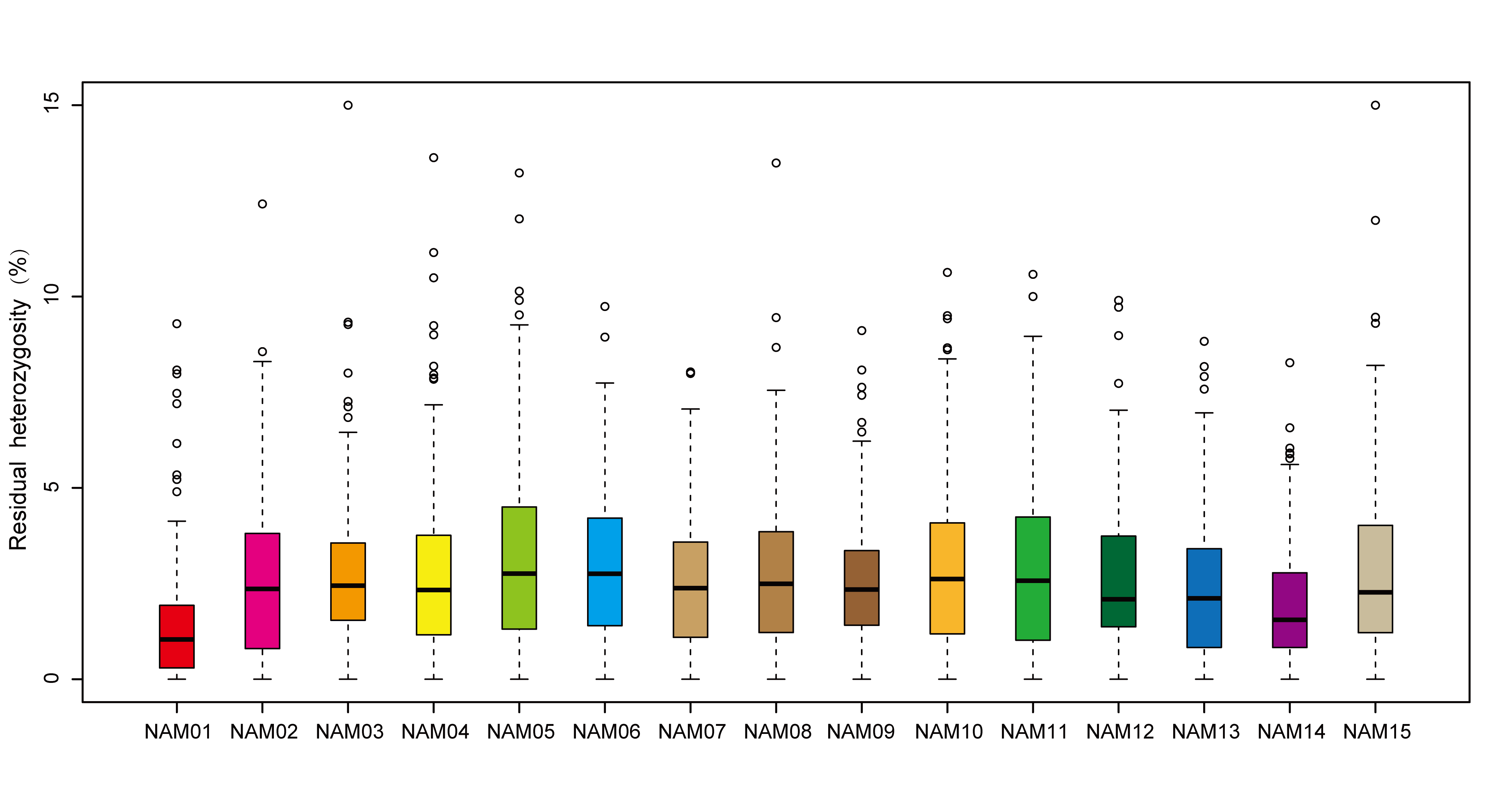

Supplement: FIGURE S5 — Boxplot of residual heterozygosity for each line in individual RIL family. [file Image_5.TIF]

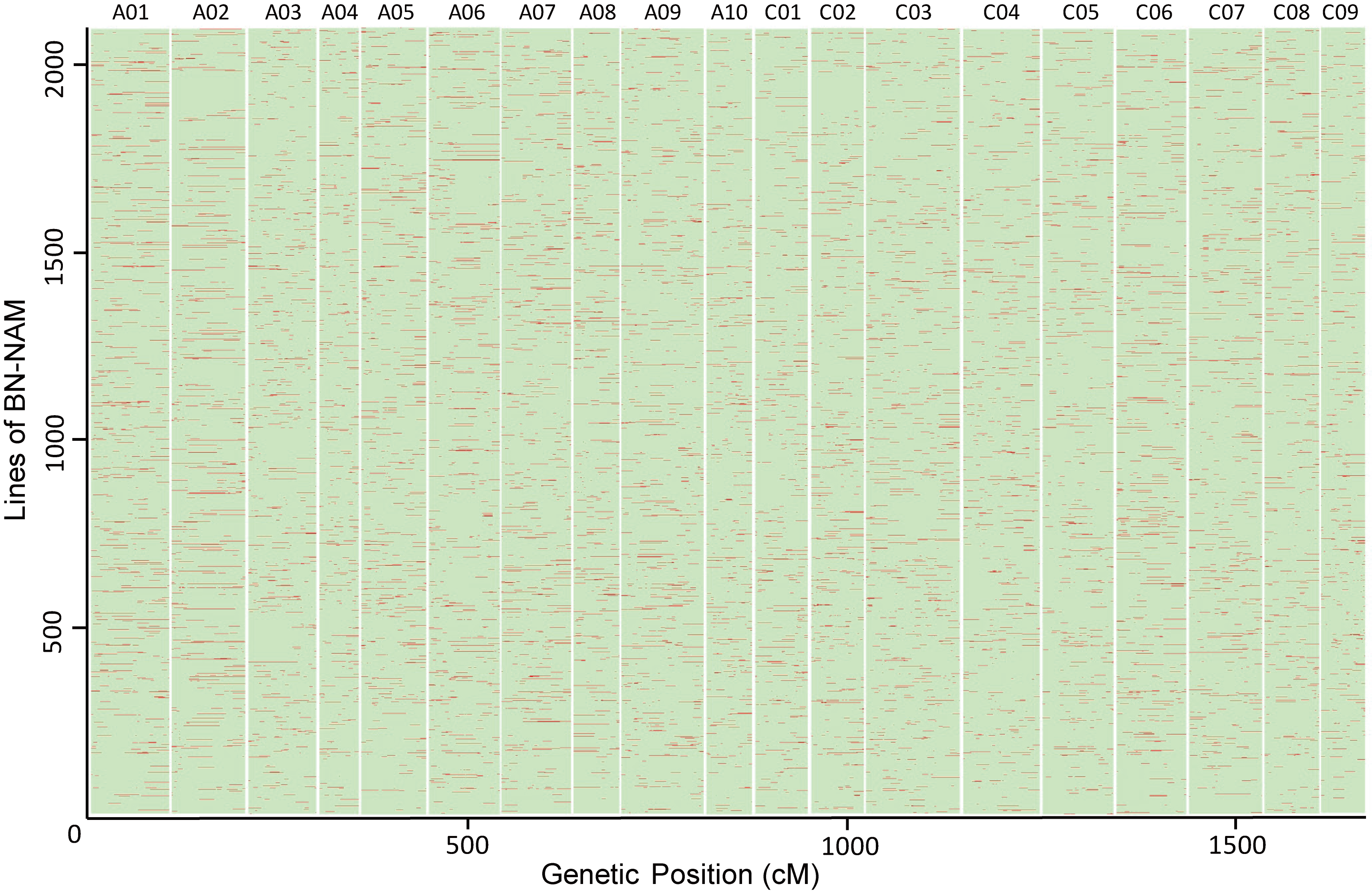

Supplement: FIGURE S6 — Residual heterozygous regions along the 19 chromosomes in all lines of the BN-NAM population. [file Image_6.TIF]
